# Machine learning to predict overall short-term mortality in cutaneous melanoma

**DOI:** 10.1007/s12672-023-00622-5

**Published:** 2023-01-31

**Authors:** C. Cozzolino, A. Buja, M. Rugge, A. Miatton, M. Zorzi, A. Vecchiato, P. Del Fiore, S. Tropea, A. Brazzale, G. Damiani, L. dall’Olmo, C. R. Rossi, S. Mocellin

**Affiliations:** 1 Soft-Tissue, Peritoneum and Melanoma Surgical Oncology Unit, Veneto Institute of Oncology IOV-IRCCS, Via Gattamelata, 64, 35128 Padua, PD Italy; 2 Department of Cardiac, Thoracic, Vascular Sciences, and Public Health, University of Padua, Padua, Italy; 3 Veneto Tumor Registry (RTV), Azienda Zero, Padua, Italy; 4 Pathology and Cytopathology Unit, Department of Medicine - DIMED, University of Padua, Padua, Italy; 5 Department of Statistical Sciences, University of Padua, Padua, Italy; 6 Clinical Dermatology, IRCCS Istituto Ortopedico Galeazzi, Milan, Italy; 7 Department of Surgery, Oncology and Gastroenterology - DISCOG, University of Padua, Padua, Italy

**Keywords:** Artificial intelligence, Machine learning, Melanoma, Oncology, Mortality, Predictors

## Abstract

**Background:**

Cutaneous malignant melanoma (CMM) ranks among the ten most frequent malignancies, clinicopathological staging being of key importance to predict prognosis. Artificial intelligence (AI) has been recently applied to develop prognostically reliable staging systems for CMM. This study aims to provide a useful machine learning based tool to predict the overall CMM short-term survival.

**Methods:**

CMM records as collected at the Veneto Cancer Registry (RTV) and at the Veneto regional health service were considered. A univariate Cox regression validated the strength and direction of each independent variable with overall mortality. A range of machine learning models (Logistic Regression classifier, Support-Vector Machine, Random Forest, Gradient Boosting, and k-Nearest Neighbors) and a Deep Neural Network were then trained to predict the 3-years mortality probability. Five-fold cross-validation and Grid Search were performed to test the best data preprocessing procedures, features selection, and to optimize models hyperparameters. A final evaluation was carried out on a separate test set in terms of balanced accuracy, precision, recall and F1 score. The best model was deployed as online tool.

**Results:**

The univariate analysis confirmed the significant prognostic value of TNM staging. Adjunctive clinicopathological variables not included in the AJCC 8th melanoma staging system, i.e., sex, tumor site, histotype, growth phase, and age, were significantly linked to overall survival. Among the models, the Neural Network and the Random Forest models featured the best prognostic performance, achieving a balanced accuracy of 91% and 88%, respectively. According to the Gini importance score, age, T and M stages, mitotic count, and ulceration appeared to be the variables with the greatest impact on survival prediction.

**Conclusions:**

Using data from patients with CMM, we developed an AI algorithm with high staging reliability, on top of which a web tool was implemented (unipd.link/melanomaprediction). Being essentially based on routinely recorded clinicopathological variables, it can already be implemented with minimal effort and further tested in the current clinical practice, an essential phase for validating the model’s accuracy beyond the original research context.

## Introduction

Cutaneous malignant melanoma (CMM) is one of the deadliest skin cancers due to its intrinsic biological aggressiveness and relatively high probability of misdiagnosis [1, 2]. Melanoma accounted for 5.6% of all new cancer cases in the U.S. in 2021, and its incidence has been on a steady global increase over the past few decades [3–5]. In the U.S., the median age at diagnosis is 65 years, and the median age at death from melanoma is 71 years [3, 6]. In Italy, the estimated total number of new cases of cutaneous melanoma was 14,900 (8,100 in males, 6,700 in females) in 2020, while 169,900 people are estimated to be alive following a melanoma diagnosis. CMM is the third most common malignancy among Italians aged 50 years or less [6, 7]. These numbers raise concerns about optimizing the efficacy and the efficiency of CMM management, as well as the economic impact of this disease on healthcare systems [4, 6, 8].

Despite advances in early detection and treatment, CMM continues to be a disease with highly variable outcomes. Developments in systemic adjuvant medications for stage III and stage IV melanomas are contributing to improved outcomes even for advanced melanoma patients, but there are still gaps in our ability to correctly stage melanomas [9–11]. Internationally, the prognostic assessment of CMM outcome is based on the American Joint Committee on Cancer (AJCC) melanoma staging system [2, 12].

Recent theoretical applications of various artificial intelligence (AI) algorithms in oncological research have produced promising results, which might help optimize cancer care by personalizing patient treatment [13–16]. Unlike traditional computer programming, AI does not rely on a pre-determined algorithm to produce an output, but rather analyzes input data with its associated output to process a model that can be then used to infer on similar datasets [17]. In order to work properly, these types of algorithm need large amounts of data to be trained on, with the further proviso that the data must be of good quality [18]. The main advantage of AI over traditional approaches lies in its ability to analyze multiple measures in complex and large data sets, combining information, and weighing the relative impact in relation to the target outcomes, therefore offering more advanced prognostic capabilities compared to human-based staging systems [19, 20]. Given the recent increase in the development of AI-based algorithms for medical use, and the appearance in public health of good-quality, large clinical databases, the times are ready for testing the application of artificial intelligence techniques to CMM staging systems as well [13–16]. A number of significant published studies [21–25] demonstrated the effectiveness of bioinformatics analysis and machine learning to address this issue alternatively to prognostic nomograms [26] for melanoma patients. Nevertheless, most of these works never turned into a real application [25]. Moreover, richer data, including medical examination results, such as whole genome sequencing, medical imaging and pathological pictures, do not always result in better models. In fact, despite high claimed accuracy, models relying on digital pathology slides and deep learning techniques (e.g. Convolutional Neural Networks) may fall into overfitting due to the high variability of the histopathological images [27]. In addition, omics data are complex to handle and genome sequencing has expensive costs [23].

It is therefore necessary to find the right compromise between model flexibility degree and data type complexity in order to minimize bias and ensure high generalizability.

Based only on plain routinely collected CMM clinicopathological variables, as recorded by the regional population-based Veneto cancer registry, this study aims to explore the consistency of AI in predicting short term overall mortality in CMM patients and then to provide a useful online tool for clinical practice [6].

## Methods

### Context

The Italian National Health System is a public service grounded in the fundamental values of universality, free access, freedom of choice, pluralism in provision, and equity. On an organization level, the health system is regionally structured and primarily supported by general taxation [28].

In 2015, the Veneto Oncology Network (ROV) published a comprehensive document based on the current national and international literature, detailing the clinical procedures for the clinical management of CMM patients [29–32]. It included procedures to be followed from the patient’s initial diagnosis to end-of-life care, as well as a detailed set of indicators to monitor consistency between recommendations and real-world clinical practice [33].

### Clinical data

The data for the analysis were sourced from the Veneto Cancer Registry (RTV), a high-resolution, population-based dataset covering the regional population (approximately 4.9 million residents), and the regional health service records. Cancer registration procedures were based on information collected from various sources (e.g., pathology reports, death certificates, and the health service’s administrative records) [6, 33].

All incident cases of invasive CMM registered by the RTV in 2015 (1,279 cases) and 2017 (1,368 cases) were included [6, 33]. The following variables were considered for this study: demographics (age and sex); histological subtypes of CMM (malignant not otherwise specified - NOS -, superficial spreading melanoma, nodular melanoma, lentigo maligna melanoma, acral-lentiginous melanoma, desmoplastic melanoma, and spitzoid melanoma); tumor site (lower limbs, upper limbs, head, hands and feet, and trunk); CMM growth phase (radial versus vertical); ulceration (absent versus present); Breslow thickness (≤ 0.75, 0.76–1.50, 1.51–3.99, ≥ 4.00 mm); CMM regression (absent versus present); tumor-infiltrating lymphocytes (TIL) (absent versus present); mitotic count (number of mitoses per mm^2^); T, N, and M 8th edition AJCC stages at diagnosis; sentinel lymph node biopsy - SLNB - (negative versus positive); SLNB maximum metastasis diameter (in mm); number of positive lymph nodes after SLNB or lymphadenectomy; and, evaluation of overall survival (OS) time truncated at 3 years from diagnosis [6, 33].

### Data preparation

Some features were not available for all subjects, particularly tumor site (96 missing), Breslow thickness (152), ulceration (168), pTNM stage [T value (63), N value (59), M value (21)], TIL (354), mitotic count (392), growth (587), and regression (759). As the missing values were evenly distributed across the subjects, it was considered preferable to proceed with imputation strategies as opposed to discarding incomplete records so as not to excessively reduce the sample and lose information. Simple feature imputation and multivariable regression were adopted to fill in missing data exploiting the complete records (excluding the survival outcome) as a training set.

Ordinal encoding was used for Breslow thickness, while one-hot encoding was used for pTNM stage (T, N, M values), sex, histological subtypes, ulceration, regression, TIL, growth, SLNB positivity, and site.

Finally, the dataset was shuffled and split into train and test sets (test size = 10%, 265 records) to estimate the algorithms’ predictive performances without biases.

### Statistical analysis

Descriptive statistics were obtained representing categorical variables as frequencies and proportions and summarising continuous numerical variables with means, medians, and minimum–maximum intervals. A univariate analysis was performed with Cox Regression to verify the strength and direction of each independent variable on CMM mortality. The correlation matrix was also calculated to check interdependencies and redundancies in the data.

**Fig. 1:**
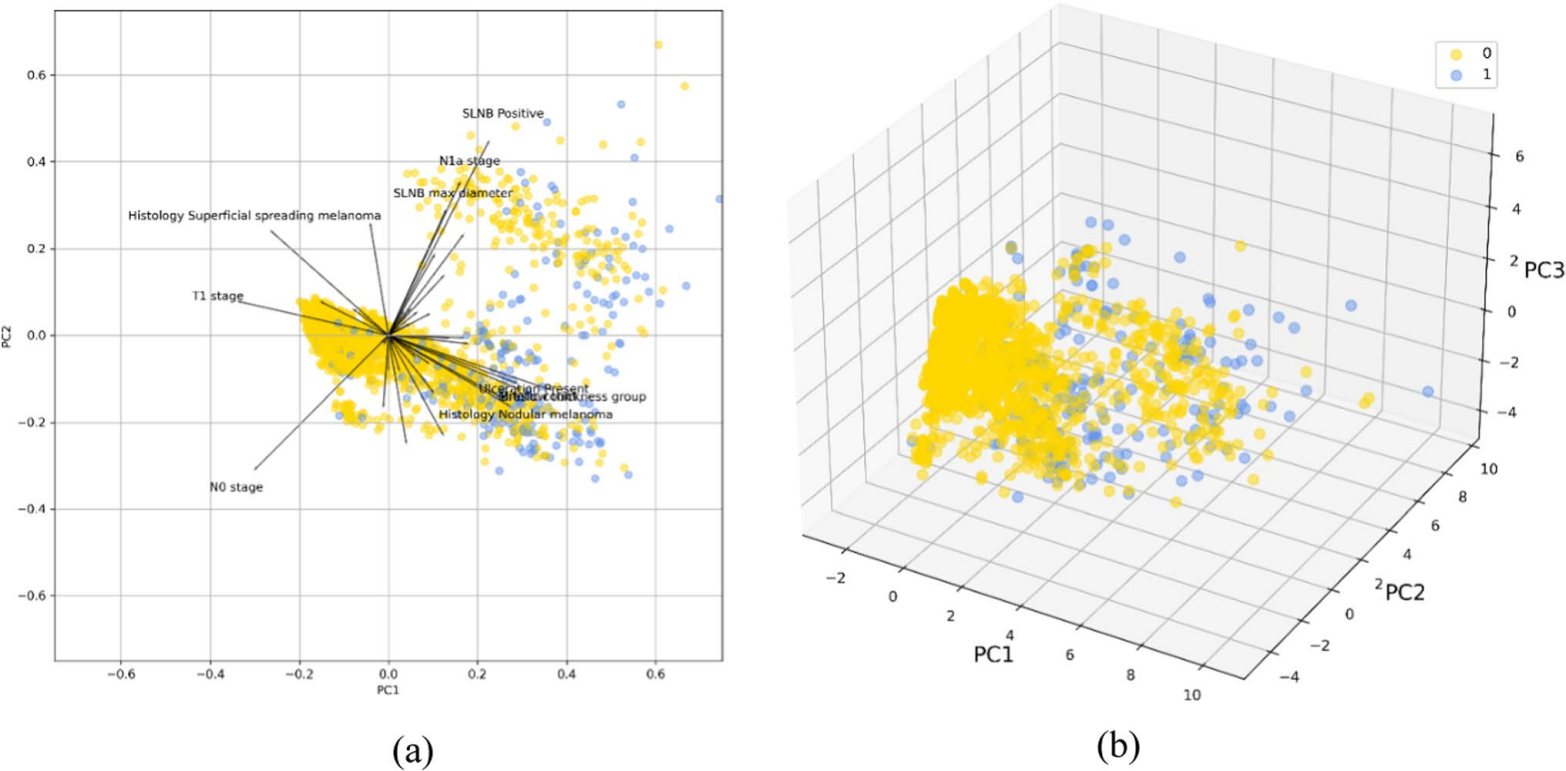
Grade of separability of the two survival classes in **a** 2 dimensions, **b** 3 dimensions principal components projections. The original features with the largest coefficients in the first two principal components are also shown (left)

Principal Components Analysis [34] was instead conducted to graphically inspect the grade of separability of the two classes, survived/deceased in our data (Fig. 1). PCA was additionally used as an alternative feature reduction strategy, selecting the minimum number of components such that the total explained variance ratio is greater than 80% of the original data.

### Machine learning models

In this study, several machine learning (ML) models were trained to predict mortality risk expressed as a binary label, survived versus deceased within 3 years from diagnosis, in a supervised learning fashion.

A range of shallow classifiers were firstly implemented with varying degrees of complexity and interpretability: Logistic Regression (LR), Support Vector Machine (SVM), Random Forest (RF), Gradient Boosting (GB), and k-Nearest Neighbors (kNN).

Logistic Regression [35] (or logit model) is a statistical technique which models the probability of an event taking place, assuming the log-odds for the event is a linear combination of one or more independent variables. Support Vector Machine [36], is a robust prediction methods that maps a set of training examples, belonging to two different classes, to points in space so as to maximise the width of the gap between the two categories. New examples are then mapped into the same space and predicted to belong to a category based on which side of the gap they fall. SVMs can efficiently be generalized for non-linear classification using the so called "kernel trick". The third model, Random Forest [37], is an ensemble-based classification or regression method that builds a large number of weak decision trees. For classification tasks, the output of the RF is the class which receives the majority of votes from the trees. Similarly, Gradient Boosting is a tree based ensemble predictor which stage-wise builds weak learners to progressively reduce the prediction error of previous model. Lastly, k-Nearest Neighbors [38] algorithm prediction is based on the shortest distance between the sample points and all of the training dataset’s points (with *k* indicating the number of nearest neighbours considered for the membership voting). As in RF, a new sample point is labelled by the class that gained the most votes.

**Fig. 2:**
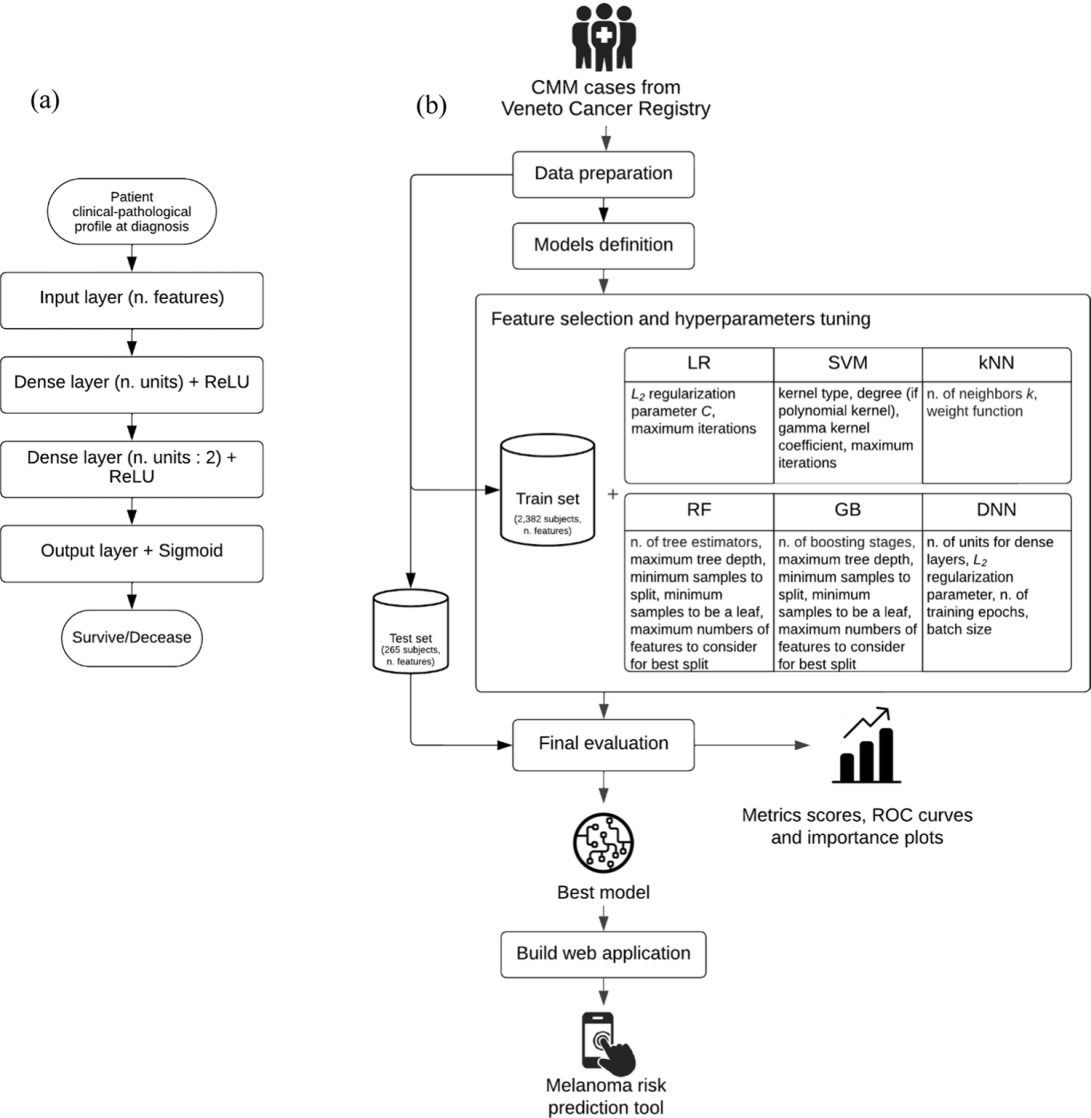
Methods overview of the study: **a** Deep Neural Network architecture diagram, **b** data and machine learning pipeline with detailed algorithms parameter settings

In this work, a simple Deep Neural Network [39] (DNN) has also been tested. The architecture, illustrated in Fig. 2a, has been designed as follows: first a standard Input layer with number of units equal to the number of features of the training data, then two dense layer with a gradually halving number of units and Rectified Linear Unit [40] activation function (ReLU), lastly a one dimensional Output layer with Sigmoid [41] activation function, since the task is binary classification. In addition, considering that DNNs are very complex and flexible models, the inner layers have been provided of *L*_*2*_ weight decay as regularization [42] technique to avoid a rapid overfitting. The training of the network weights was performed via the Adam optimizer [43] algorithm with the Binary Cross-entropy [44] as loss since the prediction task is a survived versus deceased classification.

Five-folds cross-validation [45] (CV) in combination with the Grid Search [46] optimization algorithm were performed on the training set to understand the best preprocessing procedures (feature selection, scaling, etc.) and to automatically select the best hyperparameters values between different possible combinations. The hyperparameters for each model are listed in Fig. 2b.

Given that the task of predicting CMM mortality risk is naturally defined as an imbalanced classification problem, the fitting and test evaluations were measured in terms of balanced accuracy, precision, recall and F1 score as defined below:

$$Balanced\,accuracy= \frac{1}{2}\left(\frac{TP}{P}+ \frac{TN}{N}\right)$$

$$F1=2\frac{Precision \bullet Recall}{Precision+Recall} ,$$

where

$$Precision=\frac{TP}{TP+FP}, Recall= \frac{TP}{TP+FN}$$

and *P*, *N*, *TP, TN*, *FP*, and *FN,* respectively, represent the number of real, correctly classified, and incorrectly classified examples with positive and negative classes. In this context, the positive instances (*P*) are the observed subjects deceased within 3 years from CMM diagnosis, conversely to the negative label (*N*) corresponds to survival.

Note that when running the Grid Search algorithm only the balanced accuracy metric was selected to be maximized.

R 4.0.4 was used to conduct statistical analyses. Data preparation and ML modeling were performed in Python 3.8.8, with an extensive use of “sklearn” (version 0.24.1) [47] and “tensorflow” (version 2.9.2) [48] libraries.

## Results

Table 1 depicts the distribution of the main characteristics of the CMM cohorts in the Veneto Region in 2015 and 2017. The mean follow-up duration was 1,032.8 days. The overall mortality was 10.4% at 3 years after diagnosis. The univariate analysis revealed that older age, male sex, vertical growth pattern, thicker Breslow depth, presence of ulceration, absence of TILs, higher mitotic count, SLNB positivity, wider SLNB max diameter, and greater number of positive lymph nodes are all statistically associated with short-term CMM mortality.

**Table 1:** Clinical-pathological characteristics of the study population and univariate Cox regression hazard ratio (HR) estimates and p-values

	Value	% (N = 2647)	Univariate cox regression	
			HR	*p*
Sex				
Male	1,404	53.0	1.39	0.0083
Female	1,243	47.0	r	
Age (at diagnosis), in years			1.08	< 0.0001
Mean	59.7			
Median	60			
Min–Max	15–101			
Primary site				
Trunk	1,273	48.1	1.04	0.814
Lower limb	508	19.2	r	
Upper limb	367	13.9	0.72	0.183
Head	284	10.7	1.72	0.009
Hands/feet	119	4.5	2.55	< 0.0001
CMM Histotype				
Superficial spreading	1,876	70.9	0.40	0.029
Nodular	365	13.8	2.55	0.026
Malignant (NOS)	228	8.6	2.30	0.052
Lentigo maligna	60	2.3	0.51	0.300
Spitzoid	58	2.2	0.13	0.057
Acral-lentiginous	48	1.8	r	
Desmoplastic	11	0.4	1.57	0.580
Arising from blue nevus	1	0.04	< 0.0001	0.991
Growth pattern				
Vertical	1,505	56.9	4.70	< 0.0001
Radial	555	21.0	r	
Breslow thickness, in mm				
< 0.75	1,347	50.9	r	
0.76–1.50	514	19.4	2.94	< 0.0001
1.51–3.99	381	14.4	6.14	< 0.0001
≥ 4	253	9.6	24.95	< 0.0001
Ulceration				
Absent	2,026	76.5	r	
Present	453	17.1	10.01	< 0.0001
Tumor regression				
Absent	1,160	43.8	r	
Present	728	27.5	0.42	< 0.0001
TILs				
Present	1,789	67.6	0.70	0.007
Absent	504	19.0	r	
T value				
TX	25	0.9	3.15	0.113
T0	63	2.4	26.39	< 0.0001
T1	1,612	60.9	r	
T2	370	14.0	1.83	0.031
T3	277	10.5	6.33	< 0.0001
T4	237	9.0	22.51	< 0.0001
N value				
N0	2,291	86.6	r	
N1a	125	4.7	1.47	0.196
N1b	14	0.5	10.68	< 0.0001
N1c	38	1.4	12.21	< 0.0001
N2a	47	1.8	3.46	0.0001
N2b	9	0.3	6.49	0.001
N2c	9	0.3	3.66	0.068
N3	39	1.5	17.12	< 0.0001
N3c	16	0.6	13.58	< 0.0001
M value				
M0	2,533	95.7	r	
M1	93	3.5	14.12	< 0.0001
Mitotic rate, per mm^2^			1.07	< 0.0001
Mean	2.6			
Median	1			
Min–Max	0–55			
Positive SLNB				
0 (no metastasis)	939	35.5	r	
≥ 1 (presence of metastasis)	204	7.7	2.16	< 0.0001
SLNB max diameter, in mm			1.11	0.0002
Mean	2.3			
Median	1			
Min–Max	0.03–22			
Positive Lymph Nodes			1.11	< 0.0001
Mean	0.5			
Median	0			
Min–Max	0–32			
Deceased within 3 years				
Yes	275	10.4		
No	2,372	89.6		
r = reference class				

The correlation between TNM stages and melanoma outcome was confirmed. The primary site resulted relevant when the tumor is located on the hands, feet, or head. However, upper limb or trunk localizations do not appear to have a higher hazard in comparison to lower limbs. Regarding histology, nodular and malignant (NOS) subtypes had the highest hazard ratios (HR 2.55 and 2.30, respectively; acral-lentiginous subtype as the reference category), while superficial spreading correlates with better outcomes (HR 0.40; acral-lentiginous subtype as the reference category). Desmoplastic melanoma, spitzoid melanoma, and lentigo maligna melanoma were also linked with higher survival rates, but the results were not statistically significant (p-values 0.991, 0.057, and 0.300, respectively). Similarly, the desmoplastic subtype’s HR showed an increased risk not statistically significant (p-value 0.580).

**Fig. 3:**
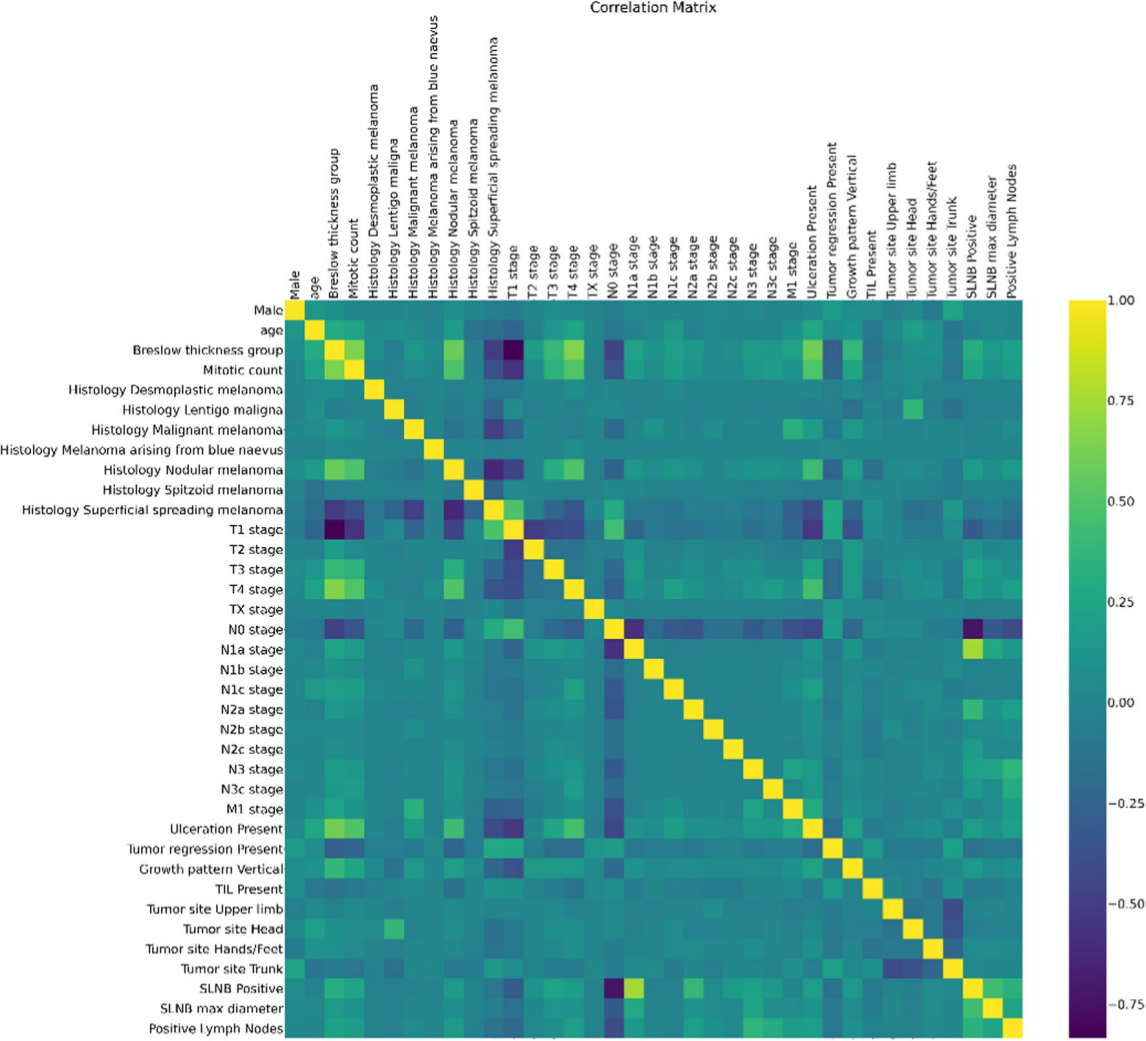
Features correlation matrix

As expected, the correlation analysis revealed interdependence between T stage values and Breslow thickness, as well as between N stages and SLNB positivity (Fig. 3). For this reason, the ML models were trained and evaluated on two different variables subsets: one excluding Breslow, number of positive lymph nodes, SLNB positivity and maximum diameter, one excluding T and N stages. A third tested alternative of feature selection was provided by PCA, which naturally reduces dimensionality and remove multicollinearity from the data at the cost of a poorer results interpretability.

**Fig. 4:**
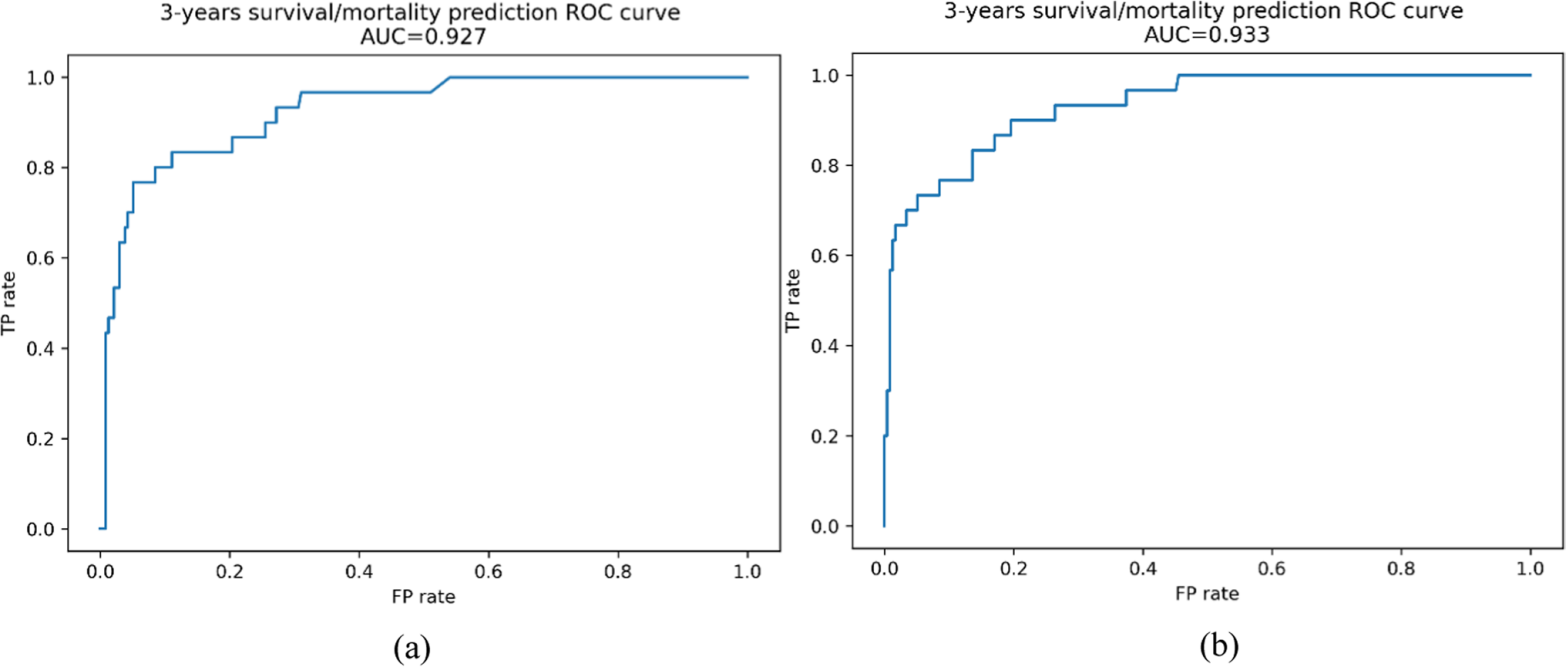
Best prognostic models ROC curves and AUC values, **a** RF, **b** DNN

The prognostic performances of the implemented classifiers are reported in Table 2. Given its more complex structure and the known universal approximation ability, the DNN unsurprisingly outperformed the shallow predictors both in terms of balanced accuracy and F1 score. However, the ensemble models, GB and RF, have achieved only slightly worse results. With a balanced accuracy of 91.1%, respectively 88.0%, the DNN and the RF proved to be the best options. Figure 4 shows the two models’ Receiver Operating Characteristic (ROC) curves and relative area under the curve (AUC) values.

**Table 2:** ML models performances in CMM mortality risk prediction

Final evaluation					
Model	Features set*	Balanced accuracy (%)	Precision (%)	Recall (%)	F1 score (%)
LR	1	74.2	80.0	51.1	62.3
	2	73.6	86.7	49.1	62.7
	3	75.3	80.0	53.3	64.0
SVM	1	74.6	76.7	52.3	62.2
	2	72.9	83.3	48.1	61.0
	3	72.9	76.7	48.9	59.7
GB	1	81.7	40.0	70.6	51.1
	2	88.6	50.0	83.3	62.5
	3	80.8	43.3	68.4	53.1
RF	1	88.0	46.7	82.4	59.6
	2	83.6	46.7	73.7	57.1
	3	83.6	46.7	73.7	57.1
kNN	1	78.0	30.0	64.3	40.9
	2	72.4	23.3	53.8	32.6
	3	83.6	46.7	73.7	57.1
DNN	1	91.1	75.0	91.0	80.0
	2	90.9	65.0	91.0	70.0
	3	79.3	66.0	79.0	70.0

^*^1: with T and N values (33 features), 2: with Breslow thickness and lymph nodes status (23 features), 3: first 20 principal components

**Fig. 5:**
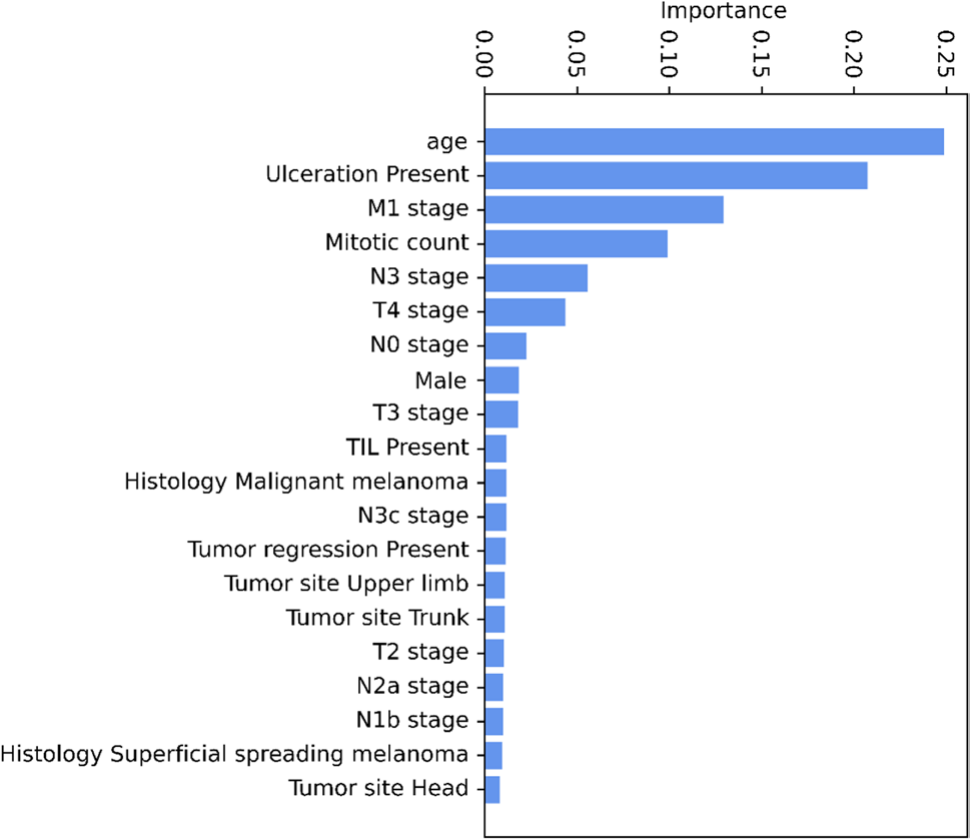
RF model most important features in predicting CMM risk according to the Gini impurity criterion

As RF is a tree-based model, it was also possible to extract each feature’s Gini importance score [49] and to represents the most important variables for CMM risk prediction (Fig. 5). The patient’s age, mitotic rate, T4 staging, the presence of ulceration, and metastasis appear to have the greatest influence on the classification of short-term mortality.

Finally, a web application was built on top of the best developed model. The tool is accessible for free at the following web page: unipd.link/melanomaprediction.

## Discussion

This study developed a machine learning algorithm that effectively predicts short-term overall mortality of patients with CMM.

In recent years, machine learning has been applied extensively to improve melanoma risk stratification and prognosis prediction. Most research has focused on finding new clinical and pathological markers [6, 10]. Nevertheless, none of the new, promising, prognostic variables have yet been added to the AJCC system, which is currently the gold standard staging system [9, 50]. Stage II and III patients currently have access to different therapeutic strategies (with or without adjuvant strategies), resulting in a subset of stage II patients having worse survival rates than stage III patients [10]. A more accurate prognostic tool is needed to increase the survival of melanoma patients by preventing recurrence and providing the most appropriate follow-up regimens [6, 10].

We decided to focus on the implementation of an algorithm based on known and validated prognostic factors, with the aim of using machine learning to improve prediction capabilities and facilitate the application of this novel melanoma risk stratification tool [6]. The results of an initial univariate analysis on the available subjects’ characteristics were consistent with those of earlier scientific literature. In addition, histological features, including thicker Breslow depth, the presence of ulceration, SLNB positivity, and the absence of TILs, are widely accepted [51–54]. In contrast, other prognostic factors, including a vertical growth pattern, a higher mitotic count, a wider diameter of the metastasis in the SLNB, a greater number of positive lymph nodes, and tumor regression (which did not reach significance in our analysis), have demonstrated less relevance and, therefore, were evaluated differently in various research contexts [51–54]. Consistently with previous research, our analysis also proved that some already known prognostic factors, namely primary site location, histology subtype, and N stage, may have different relevance depending on the specific class considered in the prognosis [51–53, 55]. These findings suggest that a better classification of existing prognostic factors is possible. [6]

The results of training a new model through machine learning are promising [6]. Using only routinely collected information, our best algorithm, a small Deep Neural Network, was able to attain an accuracy of 91.1% and an AUC value of 93.3%. Comparatively, one previous study on the prognostic accuracy of the AJCC staging system, 8^th^ edition, reported an AUC of 74% (on a cohort of 1,462 patients). [53]

One previous study by Arora et al*.* [50] tried the same approach, using the least number of routinely-used variables to produce an improved risk stratification algorithm for melanoma. They analyzed a subset of 449 patients from The Cancer Genome Atlas (TCGA) and developed an algorithm to predict life expectancy based on Breslow thickness, N staging, M staging, and ulceration status (“CMcrpred,” which is accessible via a web page and an Android app). The algorithm was validated on the same cohort of patients and was reported to perform better than the traditional AJCC staging system. Arora et al*.* also reported that clinicopathological features outperformed the use of molecular biomarkers in a combined model for melanoma. [10, 50]

Another interesting work [25] deployed two AI models, one predicting the probability of 5-year survival and the second for predicting overall survival as a regression task, as an online calculator with an interactive interface. The web page prompts the user to enter not only Breslow thickness, N staging, M staging, and ulceration status, but also the patient’s gender, age at diagnosis, tumour site, eventual recurrence type and history of previous malignancies. To develop this tool, the authors retrieved more than one hundred thousand adult subjects with cutaneous melanoma from the Surveillance, Epidemiology, and End Results (SEER) database, then experimented with several techniques, including Naïve Bayes, generalized linear model, logistic regression, decision tree, random forest, gradient boosted trees, support vector machine and deep learning. The latter was found to be the best in the prediction of 5-year survival, reporting an AUC up to 91.5% and accuracy up to 84.8%, similar with the present study’ findings.

In the Italian context, Comes and colleagues [24] combined deep learning and support vector machine with the aim of learning prognostic biomarkers from cutaneous melanoma whole-slide histological images to predict 1-year disease free survival in a binary classification fashion. After feeding their models with thousands of slide crops, manually selected and annotated by two expert histopathologists, the authors obtained an AUC value of 66.7% and an accuracy of 72.7% on the validation cohort of patients. A similar approach was proposed by Li et al*.* [23], who managed to achieve an AUC of 76.9%. The superior performances of classifiers with one-dimensional vectors, used by the present study, as input compared to the two latter studies involving instead image-based models presented, confirms how difficult it is to implement high-precision model using only imaging. In fact, despite their potential, pathology images require time spending preparations, often including manual elaboration or classification, in order to uniform the crop sizes and normalize colours. Problems of slide inconsistencies could also occur due to different stain manufacturers, staining procedures or storage [24]. In addition, AI methods injecting figures usually relies on more complex architectures, such as Convolutional Neural Networks, which have more parameters to be trained and need large sets of labelled examples. These characteristics make such models more prone to possible overfitting and more difficult to generalize and use in practice.

As reported by Triantafyllidis and Tsanas [56], the incorporation of machine learning into clinical practice requires multiple steps: (1) a retrospective validation of the algorithm on an adequately powered sample; (2) integration of the algorithm into an accessible digital tool (such as a mobile phone-based tool); (3) assessment of the tool in a real-life clinical setting; and (4) monitoring the tool’s actual implementation outside the research setting through periodic reviews of its effectiveness and safety. For these reasons, we developed a web-based application (available at unipd.link/melanomaprediction) to make the research results accessible and applicable. As already discussed, in addition to possible lack of generalizability and bias introduction, this type of technology faces the main risk of never being utilized in a real-world clinical context. These problems are commonly found in the development of machine learning and are especially relevant for algorithms constructed using data from a single center [10, 17, 57, 58]. However, we encourage physicians and institutions to cross validate our results by testing the algorithm in a real-world setting.

In the end, we conducted the research using a large cohort of patients to develop an algorithm capable of predicting melanoma mortality and providing estimates of its accuracy on a separate blinded subset of patients. Comparing our work with state-of-art models, we managed to achieve similar or better results, also showing superior performances than the AJCC staging system both in accuracy and in AUC, thus supporting the hypothesis that the use of machine learning in the field of melanoma risk stratification produces better results than traditional staging systems, as hoped. This could be a first step in bringing the power of clinical stratification to non-specialist settings, and to support physician in decision making. This result is also relevant for its implications in supporting the development of new and improved AI-based staging systems, both for melanoma and other diseases.

### Limitations

It is important to note that ML has some limitations. The ML survival probability computation is not easy to understand for most users, as the methodology used by the algorithm is complex [59]. Moreover, our cohort is population-based including subjects treated in different hospitals, which could have received heterogeneous treatments, even though the Veneto region defined standardized clinical pathways shared with all different health care institutions and based on national and international guidelines. Unfortunately, no molecular biology variables were available, and thus only clinicopathological information was imputed in our model; however, we expect that implementation of molecular profiling data will further improve the accuracy of our prediction tool. Lastly, as a final note, training models on more cohort patients’ examples would have produced even more robust and accurate predictions [17, 57].

## Conclusions

Applications based on machine learning techniques will probably reshape the future of prognosis prediction in cancer medicine. Our best model achieved satisfying prognostic performance considering routinely collected information: importantly, this algorithm appears to outperform traditional approaches relying exclusively on AJCC staging system as well as state-of-art results based on deep learning strategies and digital pathology slides. Future studies are needed to investigate whether immunohistochemical tests and molecular analyses could provide new features that improve predictivity or whether they constitute an unnecessary diagnostic delay and costs increase.

To date, few applications have been tested in the real-world environment. The tool implemented in this study shows promising results and is designed to be used with minimal effort in the current clinical setting. The real-world validation of the results achieved is a necessary step to understand the actual effectiveness of the tool and to promote this technology’s integration into everyday clinical practice.

## Data Availability

The data supporting this study’s findings are held by the Veneto Epidemiological Registry and were used under license for this work, but they are not available to the general public. These data are nonetheless available from Manuel Zorzi upon reasonable request and subject to authorization from the Veneto Epidemiological Registry (Veneto Regional Authority).

## Code availability

All code for data cleaning, analysis, and modeling is available at https://github.com/coclab/predict_melanoma_mortality.
